# Physiological responses and gut microbiota dynamics in beef cattle following transport stress syndrome with melatonin or dexmedetomidine administration

**DOI:** 10.3389/fvets.2026.1861919

**Published:** 2026-09-21

**Authors:** Yaya Jiao, Zhenhua Gao, Siyang Mu, Mingfei Fan, Haolong Wang, Zihui Cai, Cong Zhu, Yi Ding

**Affiliations:** College of Veterinary Medicine, Huazhong Agricultural University, Wuhan, China

**Keywords:** beef cattle, dexmedetomidine, gut microbiota, melatonin, transport stress syndrome

## Abstract

Beef cattle transport commonly induces stress, which may disrupt physiological homeostasis and alter the gut microbiota, thereby affecting animal welfare and production performance. The present study aimed to evaluate the effects of melatonin and dexmedetomidine on transport-induced stress. A total of 15 healthy Simmental bulls were enrolled and administered melatonin, dexmedetomidine, or physiological saline prior to a 6 h transport period (*n* = 5 per group). Blood and fecal samples were collected before transport and on days 1, 4, and 7 post-transport. Hematological parameters, biochemical indices, stress-related hormones, and oxidative stress markers were measured, and fecal samples were subjected to 16S rRNA gene sequencing analysis. The results demonstrated significant treatment × time interaction effects for red blood cell (RBC), hemoglobin concentration (HGB), adrenocorticotropic hormone (ACTH), and superoxide dismutase (SOD) (*P* < 0.05). In the melatonin (MT) group, RBC and HGB levels were significantly increased on day 7, with HGB levels exceeding those in the control (CON) group (*P* < 0.05). ACTH levels were significantly elevated in both the MT and dexmedetomidine (DEX) groups on day 7 (*P* < 0.05). In addition, SOD levels in the MT and DEX groups were higher than those in the CON group on day 7 (*P* < 0.05). Regarding the gut microbiota, the Shannon diversity index in the CON group decreased significantly on day 7 (*P* < 0.05), whereas no significant changes were observed in the MT and DEX groups. Beta diversity analysis revealed a significant separation in microbial community structure among groups (*P* < 0.05). At the genus level, an increase in *Ruminococcus* was observed in the DEX group on day 7, while *Bacteroides* abundance decreased in the MT group (*P* < 0.05). These findings suggest that melatonin and dexmedetomidine may exert regulatory effects on transport-induced stress and gut microbiota; however, further studies are required to validate these effects.

## 1 Introduction

During transportation, beef cattle are exposed to various stressors such as environmental changes and transport conditions, which can lead to both physiological and psychological responses. These stress-induced reactions result in immunosuppression and may trigger pathological changes in the respiratory and digestive systems, as well as systemic disorders. This condition is known as transport stress syndrome (1, 2). In modern beef cattle production, effective stress management for newly introduced cattle is crucial for their subsequent fattening. Following transportation stress, cattle may exhibit both gut microbiota dysbiosis and symptoms of bovine respiratory disease (BRD). The association between transportation stress and the incidence of BRD is well established (3), a condition historically referred to as shipping fever (4). These health issues reduce feed efficiency, slow growth rates, impair meat production performance, and, in severe cases, result in mortality, causing significant economic losses to the beef industry (5). Transportation stress in beef cattle has been a key focus in animal husbandry research. In recent years, with growing awareness of animal welfare, significant progress has been made in alleviating transportation stress. Strategies such as optimizing transport conditions—including appropriate stocking density, installing non-slip flooring, scheduling transport at optimal times, and closely monitoring cattle health have been explored. Injection of antioxidant components such as vitamin C can enhance the immune defense capacity of beef cattle, thereby effectively alleviating the physiological responses induced by stress (6).

Melatonin is a natural hormone secreted by the pineal gland to regulate circadian rhythms. Its metabolism relies mainly on the hepatic cytochrome P450 enzyme system, with a half-life ranging from approximately 30 min to 2 h, and its final metabolites are primarily excreted in the urine (7). An increasing body of research highlights the crucial roles of melatonin in regulating physiological rhythms, cardiovascular activity, gut microbiota, and growth and development in animals (8–10). Studies suggest that this hormone may enhance the psychophysiological performance of military personnel, particularly by regulating cardiovascular fluctuations and managing stress (11). Notably, melatonin has been extensively studied for its role in modulating seasonal reproductive activities and improving reproductive performance (9). Through mechanisms such as enhancing antioxidant capacity, regulating vascular tone, and influencing gut microbiota, melatonin positively impacts immune function, nutrient absorption, and overall production efficiency. In beef cattle production, transportation and environmental changes disrupt their biological rhythms (12). Melatonin helps mitigate stress responses by regulating circadian rhythms and facilitating adaptation to environmental changes. Melatonin protects mitochondria from oxidative and nitrogen radical damage. Melatonin and its metabolites also act as potent free radical scavengers, exhibiting significant antioxidant effects. Under stress, excessive free radicals are generated in the body, and melatonin effectively scavenges these radicals (13), reducing oxidative stress-induced damage. Transportation stress also suppresses immune function, while melatonin enhances immune cell activity, improving immune responses and alleviating stress-related effects (14).

Dexmedetomidine is a highly selective α_2_-adrenergic receptor agonist. It primarily exerts its effects by activating the locus coeruleus and inhibiting norepinephrine release, thereby reducing neural excitability (15) and exerting sedative and anxiolytic effects. Dexmedetomidine has been shown to suppress the hypothalamic-pituitary-adrenal (HPA) axis and reduce cortisol secretion (16, 17). Clinical studies have demonstrated that postoperative intravenous administration of dexmedetomidine can attenuate inflammatory responses and lower stress levels. Mechanistically, dexmedetomidine activates α_2_-adrenergic receptors, which inhibits glycogen synthase kinase 3β, thereby activating the mitogen-activated protein kinase phosphatase 1 and nuclear factor erythroid 2-related factor 2 antioxidant signaling pathway and suppressing the p53-mediated apoptotic pathway. These effects significantly alleviate lipopolysaccharide-induced hepatic injury in rats (18). Moreover, dexmedetomidine has been reported to mitigate acute stress-induced liver damage by activating mitogen-activated protein kinase phosphatase 1, which in turn inhibits the nuclear factor κB signaling pathway and cellular apoptosis (19). In cardiomyocytes, dexmedetomidine exerts protective effects by blocking the toll-like receptors 4- myeloid differentiation primary response 88- nuclear factor-kappa B signaling cascade, reducing the production of proinflammatory cytokines and alleviating myocardial injury (20). Dexmedetomidine also contributes to the stabilization of hormone levels within the nervous system, which helps maintain normal metabolic functions and may enhance immune cell activity, improving resistance to potential pathogens and reducing the occurrence of stress-related syndromes. Its metabolism primarily relies on the hepatic cytochrome P450 enzyme system, particularly cytochrome P450 family 2 subfamily A member 6 and cytochrome P450 family 2 subfamily C member 19, with a half-life of 2–3 h, and the majority of its metabolic products are excreted in urine (7).

Studies have shown that transportation in cattle alters the distribution of neutrophils (NEU) and lymphocytes (LYM) in peripheral blood circulation, thereby disrupting normal immune signaling and leading to impaired immune function (21). Stanger et al. reported that road transportation lasting up to 72 h significantly reduced the numbers of white blood cells (WBC) and lymphocytes in cattle, with a recovery period of approximately six days required for these parameters to return to baseline levels (22). During stress exposure, the HPA axis is activated (23), and adrenocorticotropic hormone (ACTH) stimulates the release of corticosteroids from the adrenal cortex to alleviate the stress response of the organism (24). The gut microbiota plays a critical role as a protective barrier in maintaining host health, closely linked to immune modulation and metabolic processes. Stressors associated with transportation can disrupt the composition of the gut microbial community, thereby impairing microbial homeostasis and compromising host physiological functions (25).

In this study, we integrated hematological and biochemical measurements with 16S rRNA gene sequencing to compare blood parameters and gut microbiota changes among treatment groups. Under the conditions of this experiment, we explored the associations of melatonin and dexmedetomidine administration with transport-related physiological responses and gut microbial dynamics in beef cattle, providing preliminary evidence to support further evaluation of these interventions for transport-stress management and potential welfare-related outcomes. We hypothesized that administration of melatonin or dexmedetomidine prior to transport would be associated with attenuated transport-related physiological stress responses and alterations in gut microbiota diversity and community structure in beef cattle.

## 2 Materials and methods

### 2.1 Experimental animals

Fifteen clinically healthy, 1 year old Simmental bulls with gentle temperament and similar initial body weight were randomly selected from the same farm, with an average body weight of 479.1 ± 12.7 kg. The animals were randomly assigned to three groups (n = 5 per group): a control (CON) group receiving physiological saline, a melatonin-treated (MT) group receiving melatonin (0.36 mg/kg; dissolved in 2.5% ethanol) (26), and a dexmedetomidine-treated (DEX) group receiving dexmedetomidine (2.5 μg/kg) (27). The administration volume was 2 mL for both the MT and DEX groups, while the CON group received an equal volume of normal saline. All injections were administered intramuscularly prior to transport. After injection, animals were randomly allocated to two transport vehicles, with animals from each treatment group distributed across the two vehicles. On October 15, 2024, the experimental cattle were transported from Zhumadian, Henan Province. Each group was administered the corresponding treatment prior to transportation. After 6 h of transportation covering approximately 500 km, the cattle safely arrived in Huanggang, Hubei Province, in the evening. Two single-deck trucks (9.6 m in length) were used. Cattle were randomly loaded onto the vehicles, with a state density of 2.8 square meters per animal. The highest temperature in Zhumadian is 28 °C, the lowest temperature is 14 °C, and the relative humidity is approximately 89%. The highest temperature in Huanggang is 25 °C, the lowest is around 19 °C, and the relative humidity is approximately 88%. All cattle had access to feed and water before loading and immediately after unloading; no feed or water was provided during transport. The animal handling plan was reviewed and approved by the Ethics Committee of Huazhong Agricultural University (HZAUCA-2025-0047).

### 2.2 Sample collection and storage

To assess the physiological effects of melatonin and dexmedetomidine, blood and rectal fecal samples were collected from the same batch of cattle at four time points: before transport (D0), and on days 1 (D1), 4 (D4), and 7 (D7) after transport (28). All sampling procedures were initiated at 08:00 a.m.

Blood samples were collected from the coccygeal vein of each animal in all groups at four time points and transferred into anticoagulant tubes containing EDTA or lithium heparin. Samples in lithium heparin tubes were centrifuged at 3,500 rpm for 15 min to separate plasma, which was then stored at −80°C for the analysis of biochemical parameters, hormones, and oxidative stress markers. Blood samples in EDTA tubes were stored at 4°C for hematological examination. In addition, the rectal fecal samples of all experimental cattle were stored in 50 mL centrifuge tubes and placed at −80 °C for 16S rRNA analysis.

### 2.3 Hematological indices measurement

Hematological parameters were measured using the BC-5000Vet veterinary automatic hematology analyzer (Shenzhen Mindray Bio-Medical Electronics Co., Ltd., Shenzhen, China). The following indicators were assessed: white blood cell (WBC); NEU; LYM; monocytes (MON); eosinophils (EOS); red blood cell (RBC); hemoglobin concentration (HGB); mean corpuscular hemoglobin (MCH) and platelets (PLT) (29).

### 2.4 Plasma biochemical indices measurement

Biochemical parameters were measured using the BS-240Vet automatic biochemical analyzer (Shenzhen Mindray Bio-Medical Electronics Co., Ltd., Shenzhen, China). The following indicators were analyzed: total protein (TP) (148624008, Shenzhen Mindray Bio-Medical Electronics Co., Ltd., Shenzhen, China), albumin (ALB) (148324009, Shenzhen Mindray Bio-Medical Electronics Co., Ltd., Shenzhen, China), glucose (Glu-G) (141524011, Shenzhen Mindray Bio-Medical Electronics Co., Ltd., Shenzhen, China), aspartate aminotransferase (AST) (140225004, Shenzhen Mindray Bio-Medical Electronics Co., Ltd., Shenzhen, China), alanine aminotransferase (ALT) (140124024, Shenzhen Mindray Bio-Medical Electronics Co., Ltd., Shenzhen, China) (28). All reagents used in the analysis were supplied by the instrument manufacturer.

### 2.5 Detection of blood hormones and oxidative factors

Enzyme-linked immunosorbent assay (ELISA) was performed to determine the plasma concentrations of cortisol (COR) (MM-063801, Jiangsu Meimian Industrial Co., Ltd., Jiangsu, China), ACTH (MM-3340602, Jiangsu Meimian Industrial Co., Ltd., Jiangsu, China), catalase (CAT) (MM-5046301, Jiangsu Meimian Industrial Co., Ltd., Jiangsu, China),and superoxide dismutase (SOD) (MM-3475801, Jiangsu Meimian Industrial Co., Ltd., Jiangsu, China) in the experimental cattle (30). Commercially available assay kits were purchased from Jiangsu Meimian Industrial Co., Ltd., and all procedures were conducted strictly in accordance with the manufacturer's instructions.

### 2.6 Microbial diversity analysis

#### 2.6.1 Fecal sample DNA extraction

Genomic DNA was extracted from rectal fecal samples collected from each group of cattle. DNA quality was assessed by 1% agarose gel electrophoresis, while DNA concentration and purity were determined using a NanoDrop 2000 spectrophotometer (Thermo Scientific, Waltham, Massachusetts, United States).

#### 2.6.2 Amplification and sequencing library construction

The previously extracted DNA was used as a template for amplification with barcode-tagged specific primers 27F 5′-AGRGTTYGATYMTGGCTCAG3′) and 1492R 5′-RGYTACCTTGTTACGACTT3′) (31), targeting the full-length 16S rRNA gene. PCR amplification was performed using an ABI GeneAmp^®^ 9700 PCR system (Thermo Scientific, Waltham, Massachusetts, United States), with three independent replicates for each sample. The amplification products were then validated for quality by 2% agarose gel electrophoresis and purified using magnetic beads. After the purified product was quantified, according to the sequencing depth requirements, the corresponding amount of purified product was mixed to prepare for subsequent sequencing.

Sequencing libraries were constructed using the SMRTbell prep kit 3.0 (Pacifc Biosciences, Menlo Park, California, United States), following these steps: (1) DNA molecules were repaired, (2) DNA ends were repaired, and (3) adapters were ligated. Sequencing was performed on the PacBio Sequel IIe System platform (Majorbio Pharmaceutical Technology Co., Ltd., Shanghai, China). High-quality HiFi reads were then generated from the obtained subreads using the CCS module in SMRT-Link v11.0 software for subsequent data analysis.

#### 2.6.3 Sequencing data analysis

According to the barcode sequence, each sample data was distinguished, and only 1000-1800 bp bacterial sequences were retained. Operational taxonomic unit (OTU) clustering was performed using UPARSE 7.1 (32, 33) (http://drive5.com/uparse/, version 7.1) with 97 % similarity as the standard to remove the chimera sequence. The number of sequences in all samples was leveled to 6000 to reduce the impact of sequencing depth on subsequent Alpha and Beta diversity analysis. Sequence alignment was conducted using the Ribosomal Database Project classifier (https://sourceforge.net/projects/rdp-classifier/, version 2.11) and the Silva 16S rRNA gene database (v138), with OTUs classified and annotated at different taxonomic levels using a confidence threshold of 0.7. The community composition at various taxonomic levels was determined. Finally, functional prediction analysis of the 16S rRNA gene was performed using Phylogenetic Investigation of Communities by Reconstruction of Unobserved States 2 (PICRUSt2) (version 2.2.0) (34).

### 2.7 Statistical analysis

Hematological data were analyzed using IBM SPSS Statistics 26 (version 26, IBM Corp., Armonk, New York, United States). Figures were generated using Origin 2024 (OriginLab Corporation, Northampton, MA, USA). A baseline-adjusted linear mixed-effects model (LMM) was used to evaluate the effects of different treatments on the dynamic changes in the measured parameters of transported Simmental cattle. For each parameter, values obtained at post-transport time points (D1, D4, and D7) were treated as the dependent variable, whereas the pre-transport baseline value (D0) was included as a covariate. Treatment group (CON, MT, and DEX), time (D1, D4, and D7), and the group × time interaction were included as fixed effects, while individual animal was included as a random effect to account for the correlation among repeated measurements within the same animal. The model was specified as:


$$\begin{array}{r} Y_{ij}=\beta_{0}+\beta_{1}{Baseline}_{i}+\beta_{2}Group_{i}+\beta_{3}Time_{j}+\\ \beta_{4}{\left(Group\times Time\right)}_{ij}+u_{i}+\varepsilon_{ij} \end{array}$$

Where *Y*_*ij*_ denotes the observed value for the *i*-th animal at the *j*-th time point; β_0_ is the intercept; β_1_-β_4_ are the regression coefficients for the fixed effects; *Baseline*_*i*_ is the baseline value of the corresponding parameter for the *i*-th animal; *Group*_*i*_ represents the treatment group effect; *Time*_*j*_ represents the time effect; (*Group*×*Time*)_*ij*_ represents the interaction effect; *u*_*i*_ is the random effect for individual animal; and ε_*ij*_ is the residual error term. Prior to fitting the linear mixed-effects models, the model assumptions were evaluated based on residual diagnostics, including residual normality and homogeneity of residual variance. When significant treatment effects, time effects, or treatment × time interactions were detected, pairwise comparisons based on estimated marginal means were performed, and the Bonferroni method was used for multiple comparison correction. All data are presented as baseline-adjusted estimated marginal means ± standard error (SE), and statistical significance was set at *P* < 0.05 for two-sided tests.

Bioinformatic analysis of the gut microbiota was performed based on 16S rRNA gene sequencing data. Raw paired-end reads were quality-filtered using fastp (v0.19.6) (35) and subsequently merged using FLASH (v1.2.11) (32). Specifically, bases with a quality score < 20 at the 3′ end were trimmed using a 50-bp sliding window. Reads shorter than 50 bp after quality filtering and reads containing ambiguous bases (N) were discarded. Paired-end reads were merged with a minimum overlap length of 10 bp, and merged sequences with an overlap mismatch ratio >0.2 were removed. Samples were identified according to the barcode and primer sequences at both ends of each read, with no barcode mismatches and a maximum of two primer mismatches allowed. Quality-filtered sequences were clustered into operational taxonomic units (OTUs) at 97% sequence similarity using UPARSE (v7.1) (33), and chimeric sequences were removed simultaneously. To minimize the effect of sequencing depth on downstream alpha- and beta-diversity analyses, all samples were rarefied to 20,000 sequences per sample. After rarefaction, the average Good's coverage remained 99.09%. Taxonomic assignment of representative OTU sequences was performed using the RDP Classifier (v2.11) (34) against the SILVA 16S rRNA database (release 138) with a confidence threshold of 70%. The microbial community composition of each sample was subsequently determined at different taxonomic levels.

All data analysis of 16S rRNA was performed on the Majorbio Bio Cloud platform (https://cloud.majorbio.com). The specific workflow is as follows: First, the mothur software (36) (http://www.mothur.org/wiki/Calculators) was used to calculate the Alpha diversity index, and the Kruskal-Wallis rank sum test was used to analyze the differences between groups. Principal Coordinate Analysis (PCoA), based on Unweighted UniFrac distance, was then employed to evaluate the similarity of microbial community structures between samples. The Analysis of Dissimilarities (ADONIS) non-parametric test was used to verify the statistical significance of the differences in community structure between groups. Finally, Linear Discriminant Analysis Effect Size (LEfSe) (37) (http://huttenhower.sph.harvard.edu/LEfSe) (LDA > 3, *P* < 0.05) was used to identify community characteristics with significant differences in the relative abundance of bacterial taxa from phylum to genus level among groups, using the Kruskal-Wallis test. For taxonomic feature, overall differences were evaluated using the Kruskal-Wallis test, followed by pairwise *post-hoc* comparisons using the default procedure implemented in the Majorbio Cloud platform, with false discovery rate (FDR) correction for multiple testing. Functional profiles were predicted from the OTU table using Phylogenetic Investigation of Communities by Reconstruction of Unobserved States and summarized into Clusters of Orthologous Groups (COG) functional categories.

## 3 Results

### 3.1 Changes in hematological parameters

Table 1 shows that RBC and HGB exhibited significant treatment × time interaction effects (RBC, *P* = 0.02; HGB, *P* = 0.03). Post hoc comparisons indicated that, in the MT group, RBC was significantly increased on D7 compared with D1 (*P* = 0.01). For HGB, the MT group showed significantly higher levels than the CON group on D7 (*P* = 0.04), and a significant increase was also observed within the MT group on D7 compared with day 1 (*P* = 0.01) (Figure 1).

**Table 1:** Effects of different treatment groups on hematological parameters in transported Simmental cattle.

Indicators	Time point	Group			Time × group		Time		Group	
		CON	MT	DEX	*F*	*P*	*F*	*P*	*F*	*P*
WBC (10^9^/L)	D1	11.14 ± 1.01	9.86 ± 1.07	11.07 ± 1.00	1.14	0.36	7.30	< 0.001	0.93	0.42
	D4	8.50 ± 1.01	8.56 ± 1.07	8.91 ± 1.00						
	D7	9.94 ± 1.01	6.55 ± 1.07	7.57 ± 1.00						
NEU (10^9^/L)	D1	5.54 ± 0.81	5.51 ± 0.84	5.53 ± 0.79	2.24	0.09	9.36	< 0.001	0.54	0.59
	D4	3.40 ± 0.81	3.85 ± 0.84	3.84 ± 0.79						
	D7	5.15 ± 0.81	2.21 ± 0.84	2.59 ± 0.79						
LYM (10^9^/L)	D1	4.81 ± 0.36	3.84 ± 0.38	4.74 ± 0.36	0.79	0.54	0.45	0.64	0.52	0.61
	D4	4.39 ± 0.36	4.33 ± 0.38	4.29 ± 0.36						
	D7	4.16 ± 0.36	4.14 ± 0.38	4.30 ± 0.36						
MON (10^9^/L)	D1	0.48 ± 0.08	0.50 ± 0.08	0.49 ± 0.08	0.49	0.74	4.08	0.03	0.52	0.61
	D4	0.43 ± 0.08	0.39 ± 0.08	0.43 ± 0.08						
	D7	0.39 ± 0.08	0.21 ± 0.08	0.36 ± 0.08						
EOS (10^9^/L)	D1	0.17 ± 0.05	0.22 ± 0.05	0.22 ± 0.05	0.67	0.62	0.67	0.52	1.36	0.29
	D4	0.14 ± 0.05	0.21 ± 0.05	0.27 ± 0.05						
	D7	0.12 ± 0.05	0.20 ± 0.05	0.23 ± 0.05						
RBC (10^12^/L)	D1	7.77 ± 0.36	7.56 ± 0.36	7.69 ± 0.35	3.41	0.02	0.85	0.44	0.80	0.47
	D4	7.45 ± 0.36	8.14 ± 0.36	7.37 ± 0.35						
	D7	7.44 ± 0.36	8.47 ± 0.36	7.60 ± 0.35						
HGB (g/L)	D1	112.48 ± 3.45	108.60 ± 3.47	111.11 ± 3.51	3.03	0.03	1.41	0.26	1.19	0.33
	D4	106.88 ± 3.45	114.20 ± 3.47	106.71 ± 3.51						
	D7	107.48 ± 3.45	120.40 ± 3.47	110.51 ± 3.51						
MCH (pg)	D1	14.17 ± 0.33	14.43 ± 0.30	14.85 ± 0.32	1.78	0.16	3.75	0.04	1.47	0.26
	D4	13.99 ± 0.33	14.17 ± 0.30	14.91 ± 0.32						
	D7	14.17 ± 0.33	14.33 ± 0.30	14.93 ± 0.32						
PLT (10^9^/L)	D1	348.91 ± 82.97	223.45 ± 86.66	273.65 ± 83.19	1.00	0.43	5.29	0.01	0.34	0.72
	D4	436.71 ± 82.97	534.85 ± 86.66	391.45 ± 83.19						
	D7	442.31 ± 82.97	348.85 ± 86.66	345.25 ± 83.19						

(1) Data are presented as least squares means ± standard error (LSM ± SE), with n = 5. Data were analyzed using a linear mixed-effects model, with D0 included as a covariate for baseline adjustment. *P* values are reported to two decimal places, and values < 0.0001 are presented as *P* < 0.001. (2) CON, control group; MT, melatonin group; DEX, dexmedetomidine group. D1, D4, and D7 represent days 1, 4, and 7 after transport, respectively. The values at D1, D4, and D7 are baseline-adjusted estimated marginal means. (3) *F* and *P* represent the *F* statistic and *P* value for the corresponding effect, respectively. *P* < 0.05 indicates statistical significance. Time × Group represents the interaction effect between time and group, Time represents the main effect of time, and Group represents the main effect of group. (4) WBC, white blood cells; NEU, neutrophils; LYM, lymphocytes; MON, monocytes; EOS, eosinophils; RBC, red blood cells; HGB, hemoglobin; MCH, mean corpuscular hemoglobin; PLT, platelets.

**Figure 1:**
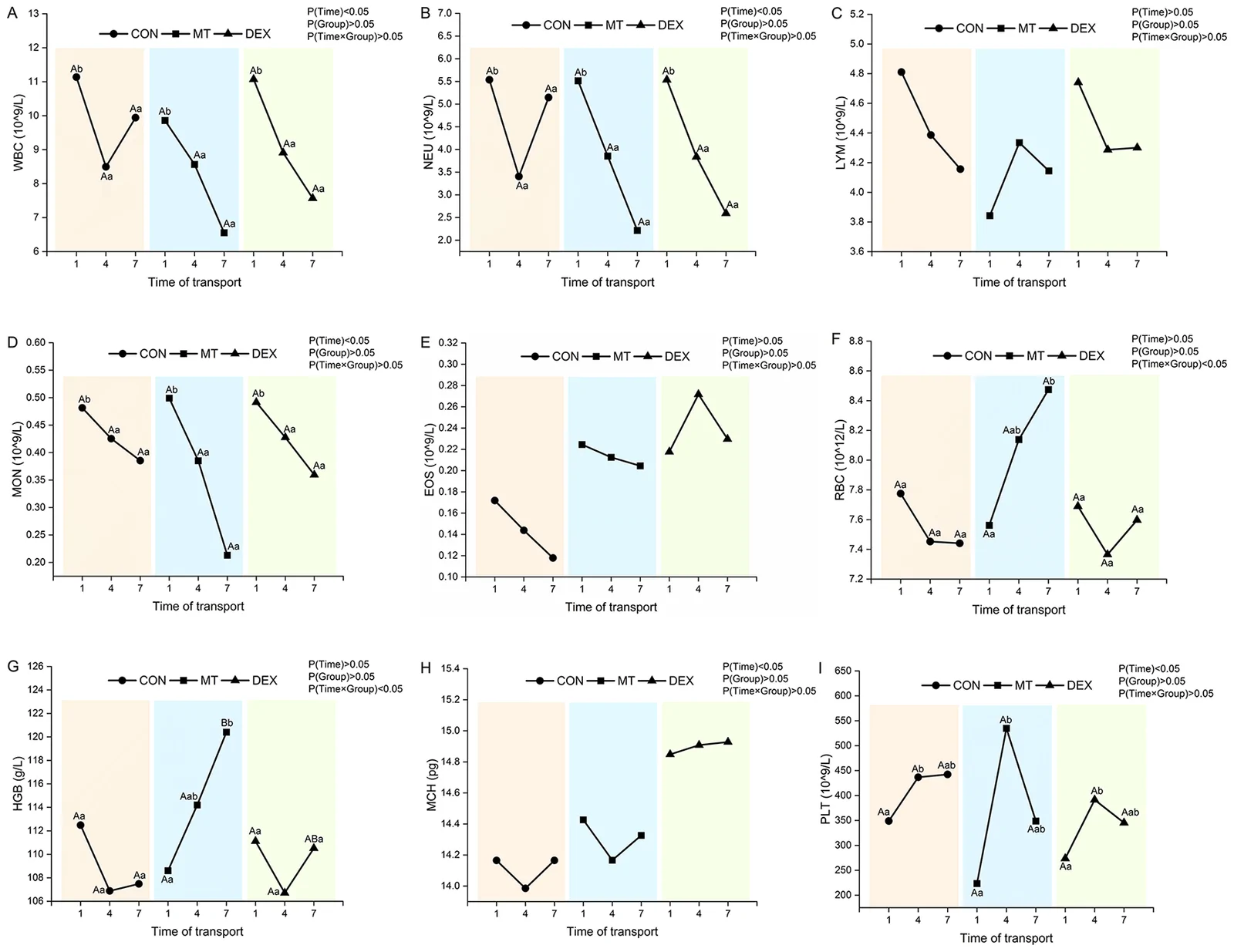
Temporal changes in hematological parameters at each time point in transported Simmental cattle across different treatment groups. **(A)** WBC, white blood cells; **(B)** NEU, neutrophils; **(C)** LYM, lymphocytes; **(D)** MON, monocytes; **(E)** EOS, eosinophils; **(F)** RBC, red blood cells; **(G)** HGB, hemoglobin; **(H)** MCH, mean corpuscular hemoglobin; and **(I)** PLT, platelets. CON = control group; MT = melatonin group; DEX = dexmedetomidine group. D1, D4, and D7 represent days 1, 4, and 7 after transport, respectively. (1) When a significant interaction effect was detected, different lowercase letters indicate significant differences among time points within the same group (*P* < 0.05), whereas different uppercase letters indicate significant differences among groups at the same time point (*P* < 0.05). When only a main effect was present, different uppercase letters indicate significant differences among groups (*P* < 0.05), and different lowercase letters indicate significant differences among time points (*P* < 0.05). Data were analyzed using a linear mixed-effects model with D0 included as a covariate for baseline adjustment, followed by post hoc multiple-comparison correction. (2) Significant time effects were observed for WBC, NEU, MON, MCH, and PLT (*P* (Time) < 0.05), although no significant differences were detected for MCH in the post hoc multiple comparisons. Significant time × group interactions were observed for RBC and HGB (*P* (Time × Group) < 0.05). No significant effects were observed for LYM or EOS.

White blood cell count, neutrophils, monocytes, mean corpuscular hemoglobin, and platelets showed no significant treatment × time interaction effects (WBC, *P* = 0.36; NEU, *P* = 0.09; MON, *P* = 0.74; MCH, *P* = 0.16; PLT, *P* = 0.43). However, significant time effects were observed for WBC, NEU, MON, MCH, and PLT (WBC, *P* < 0.001; NEU, *P* < 0.001; MON, *P* = 0.03; MCH, *P* = 0.04; PLT, *P* = 0.01). Post hoc comparisons (Figure 1) revealed that WBC levels were significantly decreased on D4 and D7 compared with D1 (D1 vs. D4, *P* = 0.03; D1 vs. D7, *P* < 0.001). Similarly, NEU levels were significantly reduced on D4 and D7 compared with D1 (D1 vs. D4, *P* = 0.01; D1 vs. D7, *P* < 0.001). MON levels were significantly decreased on D7 compared with D1 (*P* = 0.02), whereas PLT levels were significantly increased on D4 compared with D1 (*P* = 0.01). However, no significant differences were detected for MCH in the post hoc multiple comparisons (*P* > 0.05).

Lymphocytes and eosinophils showed no significant interaction effects (LYM, *P* = 0.54; EOS, *P* = 0.62), nor were significant time effects (LYM, *P* = 0.64; EOS, *P* = 0.52) or treatment effects (LYM, *P* = 0.61; EOS, *P* = 0.29) observed.

The coefficients of variation for all parameters are presented in Supplementary Table 1. Baseline comparisons indicated that no significant differences were observed in any hematological parameters among the CON, MT, and DEX groups at the start of the experiment (*P* > 0.05) (Supplementary Table 4).

### 3.2 Changes in plasma biochemical parameters

Table 2 shows that Glu-G, ALT, AST, TP, and ALB exhibited no significant treatment × time interaction effects (Glu-G, *P* = 0.11; ALT, *P* = 0.56; AST, *P* = 0.86; TP, *P* = 0.57; ALB, *P* = 0.57). Glucose showed only a significant main effect of treatment (*P* = 0.02). *Post-hoc* comparisons (Figure 2) indicated that, for Glu-G, significant differences were observed between the CON) and MT groups (*P* = 0.04), as well as between the CON and DEX groups (*P* = 0.05).

**Table 2:** Effects of different treatment groups on blood biochemical parameters in transported Simmental cattle.

Indicators	Time point	Group			Time × group		Time		Group	
		CON	MT	DEX	*F*	*P*	*F*	*P*	*F*	*P*
Glu-G (mmol/L)	D1	4.63 ± 0.18	3.83 ± 0.18	4.21 ± 0.18	2.01	0.11	0.45	0.64	4.32	0.02
	D4	4.09 ± 0.18	4.12 ± 0.18	4.04 ± 0.18						
	D7	4.48 ± 0.18	4.12 ± 0.18	3.84 ± 0.18						
ALT (U/L)	D1	36.84 ± 2.14	36.98 ± 2.14	42.10 ± 2.12	0.75	0.56	36.92	< 0.001	1.14	0.35
	D4	32.20 ± 2.14	34.08 ± 2.14	35.36 ± 2.12						
	D7	28.78 ± 2.14	27.88 ± 2.14	30.94 ± 2.12						
AST (U/L)	D1	159.65 ± 11.65	162.44 ± 11.68	178.85 ± 11.73	0.32	0.86	60.70	< 0.001	0.51	0.61
	D4	102.01 ± 11.65	113.88 ± 11.68	116.73 ± 11.73						
	D7	85.27 ± 11.65	83.30 ± 11.68	89.13 ± 11.73						
TP (g/L)	D1	77.20 ± 2.40	76.24 ± 2.39	72.14 ± 2.55	0.74	0.57	1.08	0.35	1.77	0.20
	D4	76.86 ± 2.40	81.52 ± 2.39	73.80 ± 2.55						
	D7	78.78 ± 2.40	77.88 ± 2.39	73.78 ± 2.55						
ALB (g/L)	D1	34.27 ± 0.94	33.36 ± 0.95	32.68 ± 0.94	0.74	0.57	2.39	0.11	1.75	0.21
	D4	33.79 ± 0.94	34.94 ± 0.95	32.46 ± 0.94						
	D7	33.53 ± 0.94	32.48 ± 0.95	31.26 ± 0.94						

(1) Data are presented as least squares means ± standard error (LSM ± SE), with n = 5. Data were analyzed using a linear mixed-effects model, with D0 included as a covariate for baseline adjustment. *P* values are reported to two decimal places, and values < 0.0001 are presented as *P* < 0.001. (2) CON, control group; MT, melatonin group; DEX, dexmedetomidine group. D1, D4, and D7 represent days 1, 4, and 7 after transport, respectively. The values at D1, D4, and D7 are baseline-adjusted estimated marginal means. (3) *F* and *P* represent the *F* statistic and *P* value for the corresponding effect, respectively. *P* < 0.05 indicates statistical significance. Time × Group represents the interaction effect between time and group, Time represents the main effect of time, and Group represents the main effect of group. (4) Glu-G, glucose; ALT, alanine aminotransferase; AST, aspartate aminotransferase; TP, total protein; ALB, albumin.

**Figure 2:**
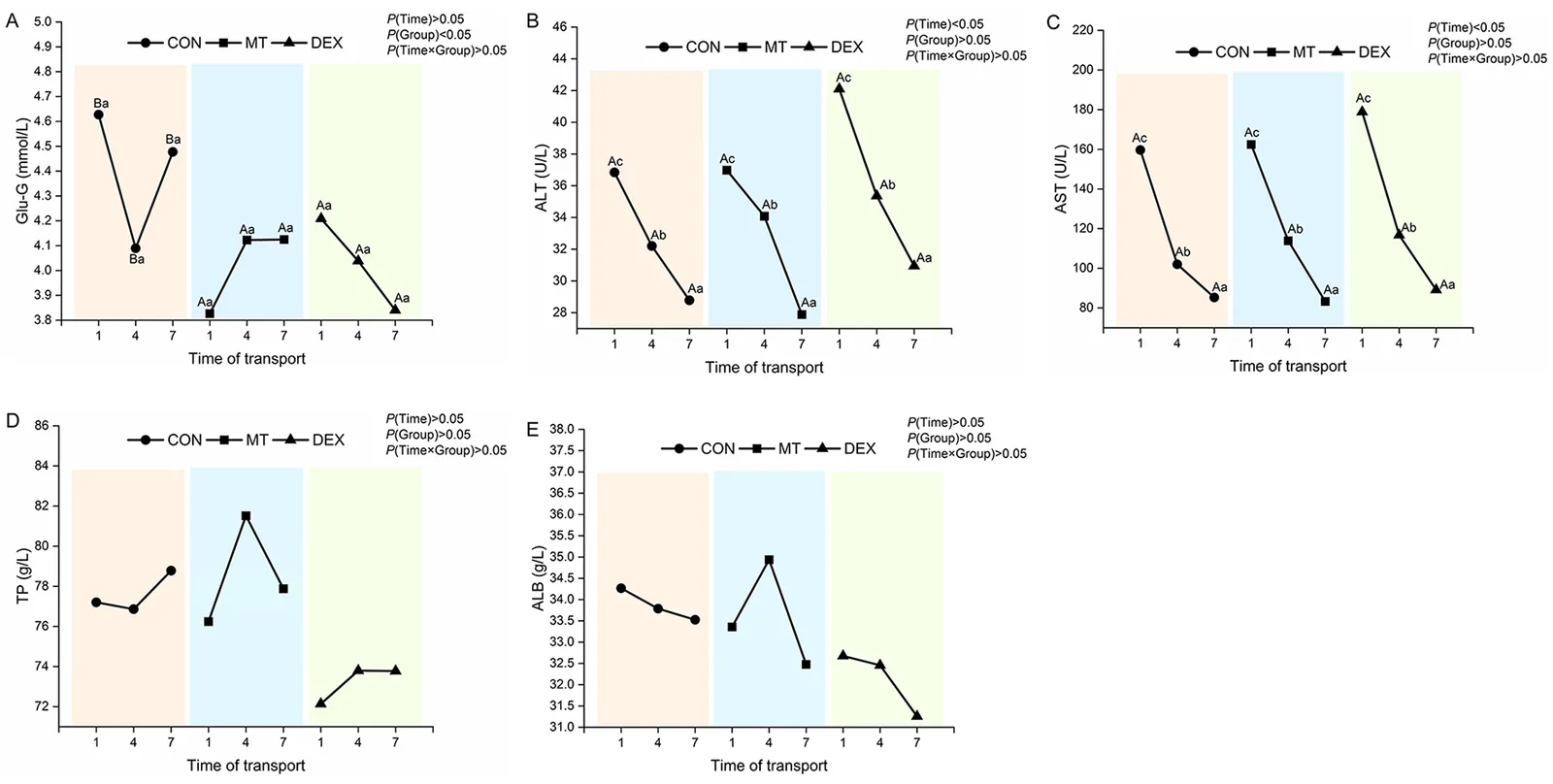
Temporal changes in biochemical parameters at each time point in transported Simmental cattle across different treatment groups. **(A)** Glu-G, glucose; **(B)** ALT, alanine aminotransferase; **(C)** AST, aspartate aminotransferase; **(D)** TP, total protein; and **(E)** ALB, albumin. CON, control group; MT, melatonin group; DEX, dexmedetomidine group. D1, D4, and D7 represent days 1, 4, and 7 after transport, respectively. (1) When a significant interaction effect was detected, different lowercase letters indicate significant differences among time points within the same group (*P* < 0.05), whereas different uppercase letters indicate significant differences among groups at the same time point (*P* < 0.05). When only a main effect was present, different uppercase letters indicate significant differences among groups (*P* < 0.05), and different lowercase letters indicate significant differences among time points (*P* < 0.05). Data were analyzed using a linear mixed-effects model with D0 included as a covariate for baseline adjustment, followed by post hoc multiple-comparison correction. (2) A significant main effect of group was observed for Glu-G (*P* (Group) < 0.05). Significant main effects of time were observed for ALT and AST (*P* (Time) < 0.05). No significant effects were observed for TP or ALB.

Alanine aminotransferase and aspartate aminotransferase showed only significant main effects of time (ALT, *P* < 0.001; AST, *P* < 0.001). *Post-hoc* comparisons demonstrated that ALT levels in all groups were highest at D1 throughout the experimental period; compared with D1, ALT levels were significantly decreased at D4 and D7 (D1 vs. D4, *P* < 0.001; D1 vs. D7, *P* < 0.001), with D7 values significantly lower than those at D4 (D4 vs. D7, *P* < 0.001). Similarly, AST levels in all groups were highest at D1; compared with D1, AST levels were significantly reduced at D4 and D7 (D1 vs. D4, *P* < 0.001; D1 vs. D7, *P* < 0.001), and values at D7 were significantly lower than those at D4 (D4 vs. D7, *P* = 0.01).

Total protein and albumin showed no significant main effects of time (TP, *P* = 0.35; ALB, *P* = 0.11) or treatment (TP, *P* = 0.20; ALB, *P* = 0.21).

The coefficients of variation for all parameters are presented in Supplementary Table 2. Baseline comparisons indicated that no significant differences were observed among the CON, MT, and DEX groups for any parameters at the start of the experiment (*P* > 0.05) (Supplementary Table 5).

### 3.3 Changes in blood hormones and oxidative factors

Table 3 shows that ACTH and SOD exhibited significant treatment × time interaction effects (ACTH, *P* = 0.046; SOD, *P* = 0.01). Further post hoc comparisons (Figure 3) indicated that, in the MT group, ACTH levels were significantly decreased at D4 compared with D1 (*P* < 0.001) and were significantly increased at D7 compared with D4 (*P* < 0.001). In the DEX group, ACTH levels were significantly increased at D7 compared with both D1 and D4 (D7 vs. D1, *P* = 0.02; D7 vs. D4, *P* < 0.001).

**Table 3:** Effects of different treatment groups on hormones and oxidative stress-related parameters in transported Simmental cattle.

Indicators	Time point	Group			Time × group		Time		Group	
		CON	MT	DEX	*F*	*P*	*F*	*P*	*F*	*P*
COR (pg/mL)	D1	434.30 ± 39.26	444.81 ± 36.81	495.72 ± 37.44	1.79	0.16	42.72	< 0.001	3.44	0.06
	D4	589.28 ± 39.26	426.29 ± 36.81	566.61 ± 37.44						
	D7	304.73 ± 39.26	240.79 ± 36.81	291.46 ± 37.44						
ACTH (pg/mL)	D1	220.39 ± 12.36	234.69 ± 12.68	213.96 ± 12.25	2.74	0.05	27.77	< 0.001	0.17	0.85
	D4	203.90 ± 12.36	182.69 ± 12.68	189.43 ± 12.25						
	D7	227.88 ± 12.36	258.91 ± 12.68	252.07 ± 12.25						
SOD (pg/mL)	D1	270.01 ± 27.80	270.18 ± 29.29	291.05 ± 26.64	3.66	0.01	8.40	< 0.001	4.25	0.02
	D4	256.81 ± 27.80	340.64 ± 29.29	249.20 ± 26.64						
	D7	109.43 ± 27.80	290.96 ± 29.29	209.86 ± 26.64						
CAT (pg/mL)	D1	362.92 ± 16.03	323.45 ± 15.90	375.73 ± 16.02	1.21	0.33	46.24	< 0.001	3.87	0.04
	D4	399.08 ± 16.03	355.33 ± 15.90	425.85 ± 16.02						
	D7	311.19 ± 16.03	285.61 ± 15.90	305.43 ± 16.02						

(1) Data are presented as least squares means ± standard error (LSM ± SE), with n = 5. Data were analyzed using a linear mixed-effects model, with D0 included as a covariate for baseline adjustment. *P* values are reported to two decimal places, and values < 0.0001 are presented as *P* < 0.001. (2) CON, control group; MT, melatonin group; DEX, dexmedetomidine group. D1, D4, and D7 represent days 1, 4, and 7 after transport, respectively. The values at D1, D4, and D7 are baseline-adjusted estimated marginal means. (3) *F* and *P* represent the *F* statistic and *P* value for the corresponding effect, respectively. *P* < 0.05 indicates statistical significance. Time × Group represents the interaction effect between time and group, Time represents the main effect of time, and Group represents the main effect of group. (4) COR, cortisol; ACTH, adrenocorticotropic hormone; SOD, superoxide dismutase; CAT, catalase.

**Figure 3:**
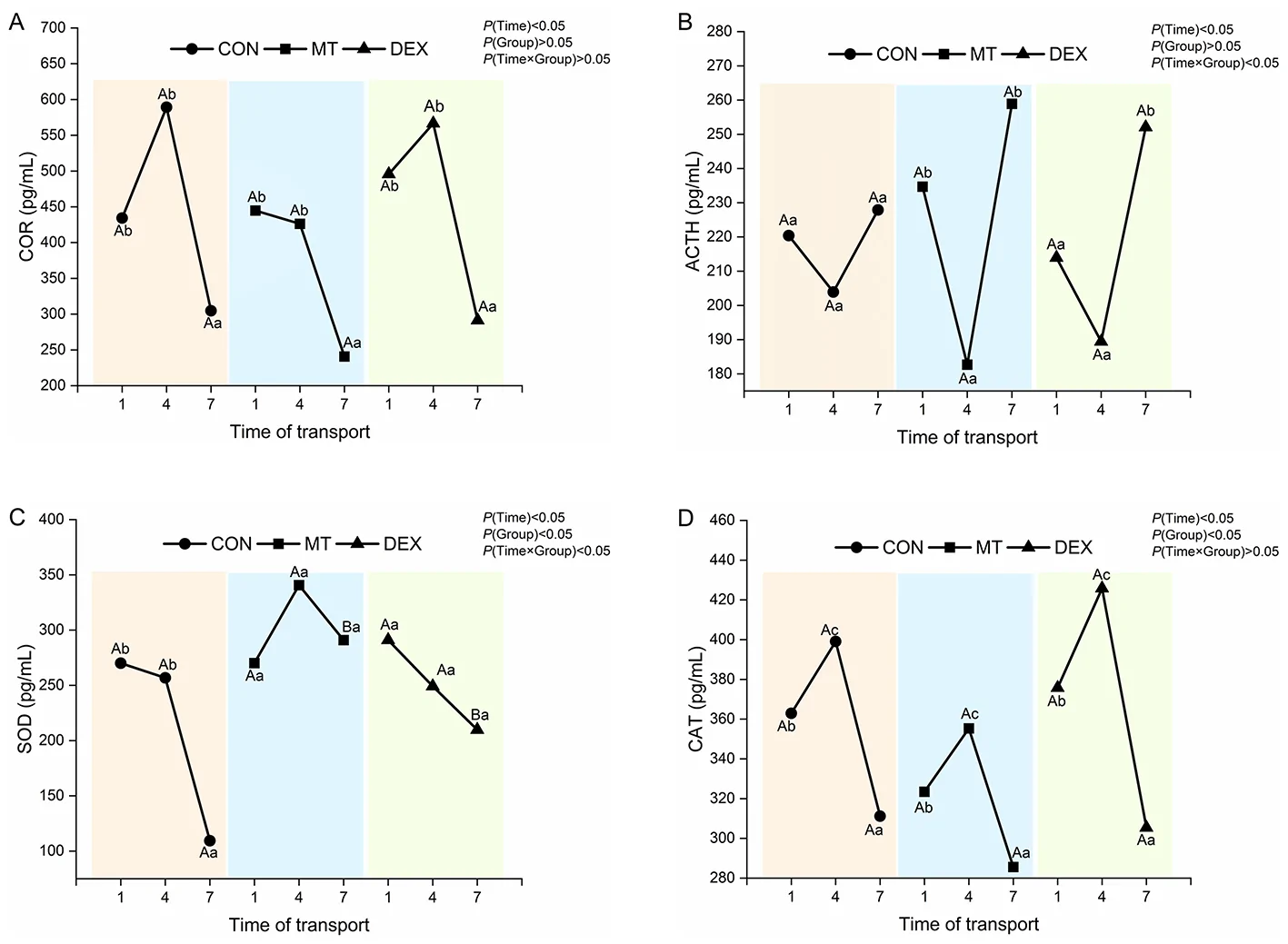
Temporal changes in hormone and oxidative stress-related parameters at each time point in transported Simmental cattle across different treatment groups. **(A)** COR, cortisol; **(B)** ACTH, adrenocorticotropic hormone; **(C)** SOD, superoxide dismutase; and **(D)** CAT, catalase. CON, control group; MT, melatonin group; DEX, dexmedetomidine group. D1, D4, and D7 represent days 1, 4, and 7 after transport, respectively. (1) When a significant interaction effect was detected, different lowercase letters indicate significant differences among time points within the same group (*P* < 0.05), whereas different uppercase letters indicate significant differences among groups at the same time point (*P* < 0.05). When only a main effect was present, different uppercase letters indicate significant differences among groups (*P* < 0.05), and different lowercase letters indicate significant differences among time points (*P* < 0.05). Data were analyzed using a linear mixed-effects model with D0 included as a covariate for baseline adjustment, followed by *post-hoc* multiple-comparison correction. (2) A significant main effect of time was observed for COR (*P* (Time) < 0.05). Significant time × group interactions were observed for ACTH and SOD (*P* (Time × Group) < 0.05). CAT showed significant main effects of both time (*P* (Time) < 0.05) and group (*P* (Group) < 0.05).

Superoxide dismutase levels at D7 were significantly higher in both the MT and DEX groups than in the CON group (MT vs. CON, *P* < 0.001; DEX vs. CON, *P* = 0.03). Within the CON group, SOD levels at D7 were significantly decreased compared with D1 and D4 (D7 vs. D1, *P* < 0.001; D7 vs. D4, *P* < 0.001).

Cortisol and catalase showed no significant interaction effects (COR, *P* = 0.16; CAT, *P* = 0.33). However, significant time effects were observed for COR and CAT (COR, *P* < 0.001; CAT, *P* < 0.001). Post hoc comparisons revealed that COR levels at D7 were significantly decreased compared with D1 and D4 (D7 vs. D1, *P* < 0.001; D7 vs. D4, *P* < 0.001). Catalase levels differed significantly across all three time points, with CAT significantly increased at D4 compared with D1, followed by a significant decrease at D7 compared with D1 (D4 vs. D1, *P* < 0.001; D7 vs. D1, *P* < 0.001; D7 vs. D4, *P* < 0.001). In addition, CAT showed a significant main effect of treatment (*P* = 0.04); however, no significant differences were detected in the post hoc multiple comparisons (*P* > 0.05).

The coefficients of variation for all parameters are presented in Supplementary Table 3. Baseline comparisons indicated that a significant difference in SOD was observed among the CON, MT, and DEX groups at the start of the experiment (*P* = 0.01), whereas no significant differences were observed for the remaining plasma hormones and oxidative stress indices (*P* > 0.05) (Supplementary Table 6).

### 3.4 Changes in gut microbiota

#### 3.4.1 Changes in gut microbiota α-diversity before and after transportation

We performed diversity analyses on 60 samples and obtained 1,573,794 quality-filtered sequences, with an average sequence length of 1,444 bp. Taxonomic annotation showed that 15 phyla were identified at the phylum level; this number increased to 245 genera at the genus level and 422 species at the species level. At the OTU level, a total of 1,290 distinct OTUs were detected.

As sequencing depth increased, the OTU-level rarefaction curves for the Shannon index, Coverage, and Sobs index gradually approached a plateau (Figures 4A–C). These results indicate that the number of OTUs captured in each fecal sample was sufficient to characterize bacterial diversity, and the current sequencing depth was adequate for downstream analyses. After transport, the Shannon index showed no significant changes in the MT and DEX groups, whereas it decreased significantly in the CON group at D7 (*P* < 0.05) (Figures 4D–F). This suggests that the α-diversity of the gut microbiota in Simmental cattle tended to decline after transport, while the magnitude of α-diversity changes was relatively small in the MT and DEX groups.

**Figure 4:**
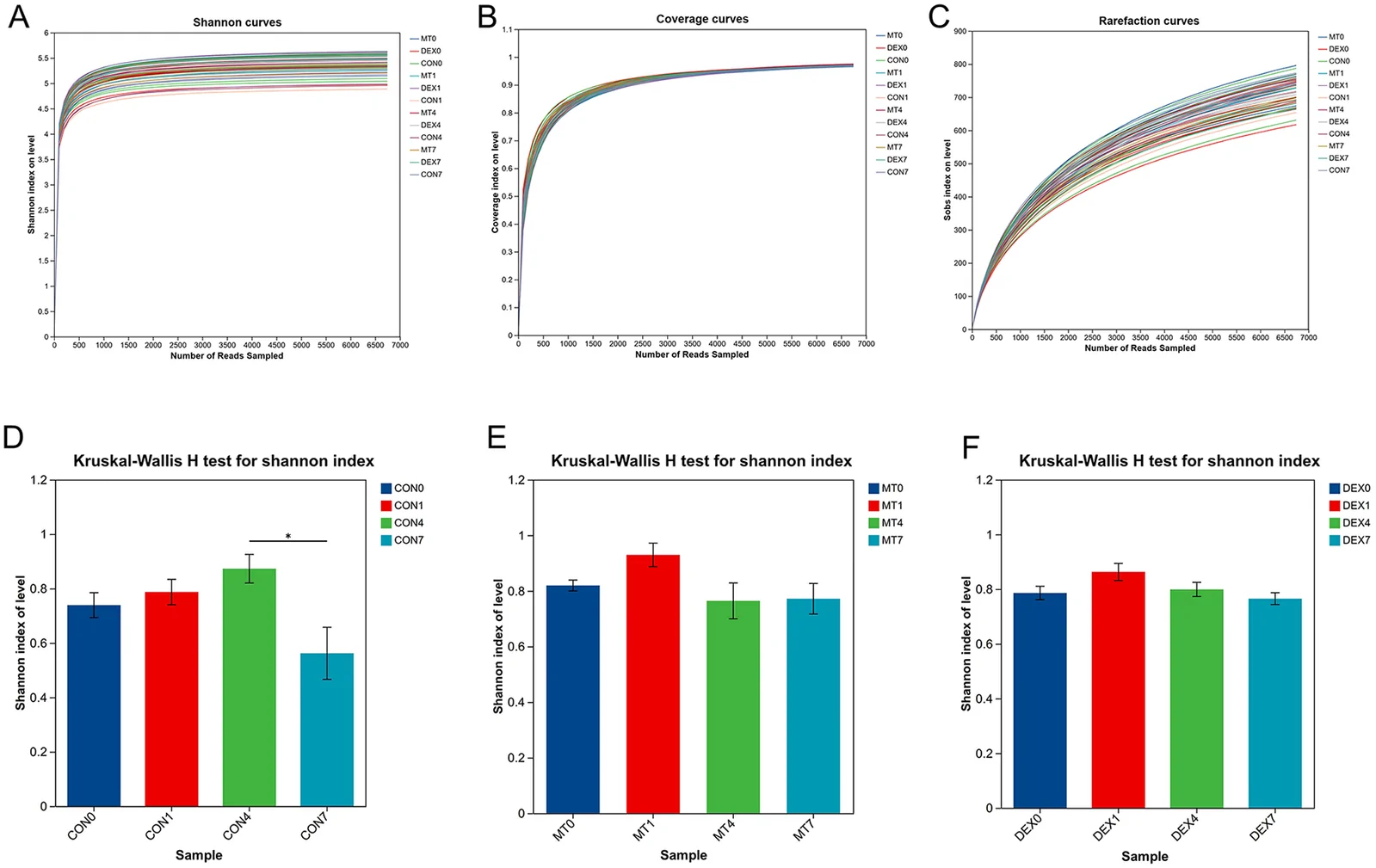
Effects of transport on the α-diversity of the gut microbiota in Simmental cattle. **(A–C)** Rarefaction curves of the Shannon index, Coverage, and Sobs index. **(D–F)** Changes in the Shannon index of gut microbial α-diversity. CON0 indicates the CON group before transport; CON1, CON4, and CON7 indicate day 1, day 4, and day 7 after arrival, respectively. The same labeling applies to the MT and DEX groups. Error bars represent the standard error of the mean. *P < 0.05.

#### 3.4.2 Changes in gut microbiota β-diversity before and after transportation

Using the Unweighted UniFrac algorithm, sample clustering trees were generated at both the OTU and genus levels. After transport, samples within the same group showed relatively high consistency in community structure, whereas clear differences in community structure were observed among groups (Figures 5A, B). Principal coordinates analysis (Figures 5C–E) indicated significant differences in community structure among the three groups at the OTU level (*P* < 0.05), showing that the microbial communities differed between the groups. At D0, the distributions of the MT, DEX, and CON groups largely overlapped, indicating similar baseline microbial community structures across groups (Figures 5F–I). At D1, partial separation among MT, DEX, and CON began to emerge. By D4, the differences among groups became more pronounced. At D7, the separation in community structure among the MT, DEX, and CON groups was significant (*P* < 0.05), with the most evident separation observed between the DEX and CON groups.

**Figure 5:**
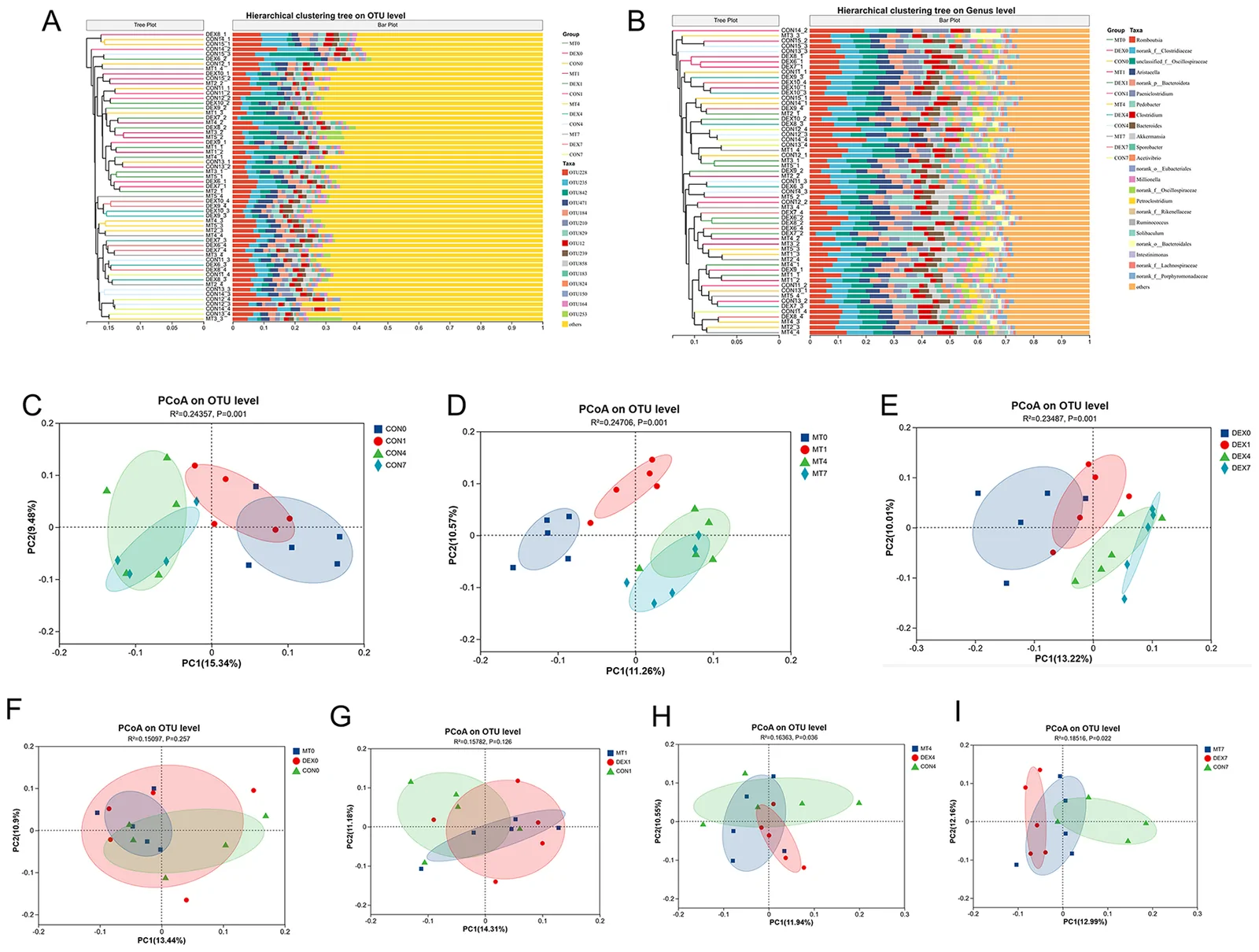
Effects of transport on the β-diversity of the gut microbiota in Simmental cattle. **(A, B)** Sample clustering trees based on Unweighted UniFrac distances at the OTU and genus levels. **(C–I)** Principal coordinates analysis plots of the gut microbiota at the OTU level in Simmental cattle before and after transport. CON0 indicates the CON group before transport; CON1, CON4, and CON7 indicate day 1, day 4, and day 7 after arrival, respectively. The same labeling applies to the MT and DEX groups.

#### 3.4.3 Analysis of the gut microbiota composition before and after transportation

*Bacillota* and *Bacteroidota* showed relatively high abundances across all groups and represented the dominant phyla (Figures 6A–C). Circos analysis further illustrated changes in the composition of the dominant genera before and after transportation. Each sample was composed of multiple dominant genera, among which *Romboutsia, norank_f__Clostridiaceae, unclassified_f__Oscillospiraceae, Aristaeella*, and *norank_p__Bacteroidota* exhibited relatively high abundances (Figure 6D). Kruskal–Wallis tests showed that the relative abundances of major phyla, including *Bacillota* and *Bacteroidota*, differed significantly among groups (*P* < 0.05). At the genus level, the relative abundances of several bacterial taxa, including *Aristaeella, norank_p__Bacteroidota, Pedobacter, Clostridium*, and *Bacteroides*, differed significantly among experimental groups (*P* < 0.05) (Figures 6E, F). LEfSe analysis further identified multiple differentially enriched microbial clades across phylogenetic levels, and the discriminative taxa exhibited clustered distributions on the cladogram. Overall, these results indicate that group treatments were associated with distinct gut microbiota composition patterns (Figure 6G).

**Figure 6:**
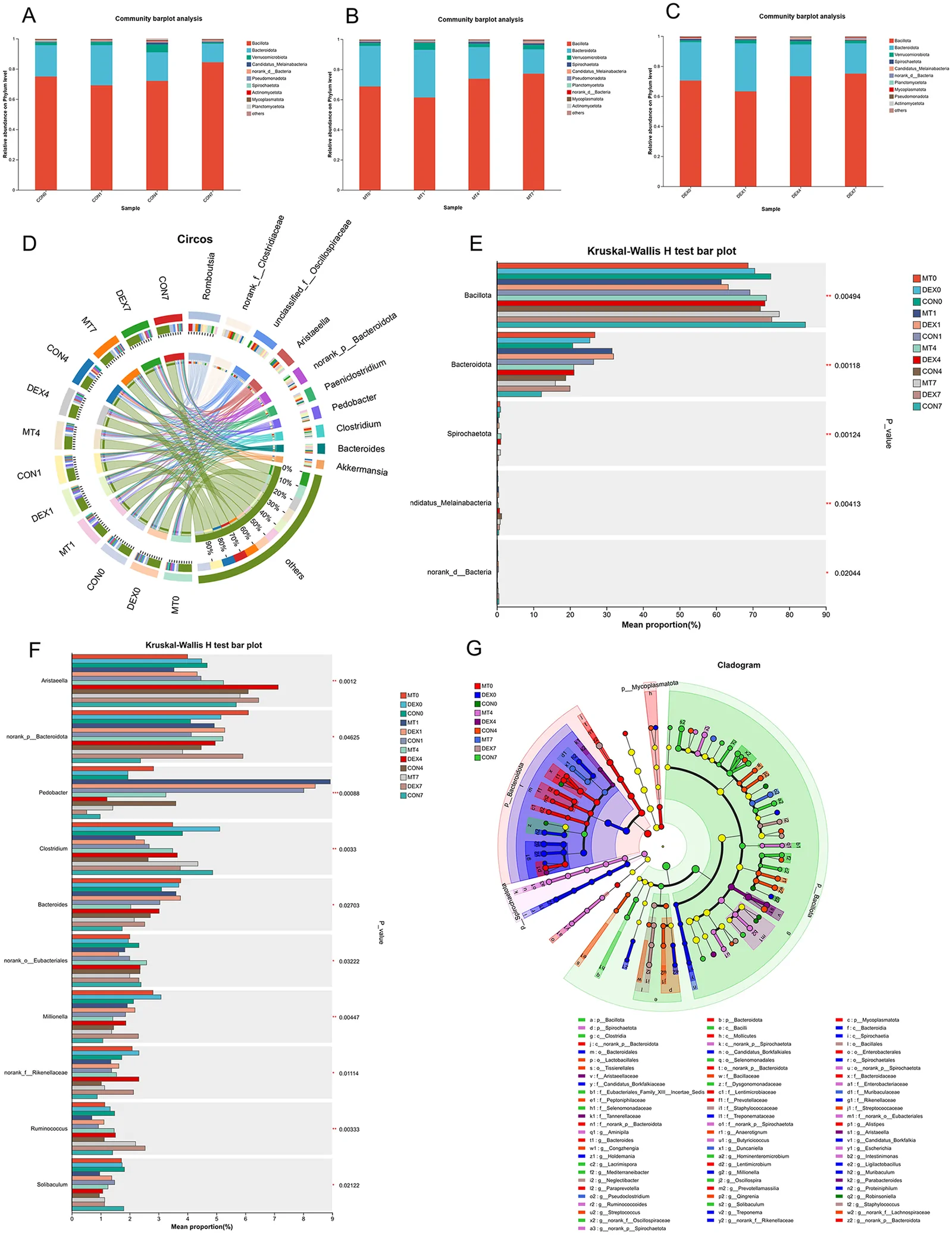
Effects of transport on the taxonomic composition of the gut microbiota in Simmental cattle. **(A–C)** Stacked bar plots showing changes in community relative abundance at the phylum level. **(D)** Circos visualization of associations between treatment groups and genus-level taxa. **(E)** Bar plot of Kruskal–Wallis tests showing between-group differences in phylum-level relative abundance. **(F)** Bar plot of Kruskal–Wallis tests showing between-group differences in genus level relative abundance. **(G)** LEfSe cladogram (phylogenetic tree) illustrating differentially abundant taxa among groups. CON0 indicates the CON group before transport; CON1, CON4, and CON7 indicate day 1, day 4, and day 7 after arrival, respectively. The same labeling applies to the MT and DEX groups.

#### 3.4.4 Analysis of the differences in gut microbiota species before and after transportation

At the phylum level, the gut microbiota of cattle was dominated by *Bacillota* and *Bacteroidota*. In the DEX group, the relative abundance of *Bacillota* increased (*P* < 0.05), whereas *Bacteroidota* decreased (*P* < 0.05) (Figures 7A, B). A similar pattern was observed in the MT group, where *Bacillota* increased over time (*P* < 0.01) and *Bacteroidota* decreased significantly (*P* < 0.01) (Figures 7C, D). Overall, both melatonin and dexmedetomidine were associated with an early post-transport increase in *Bacillota* and a transient reduction in *Bacteroidota*, followed by a gradual return of these dominant phyla toward relatively stable proportions.

**Figure 7:**
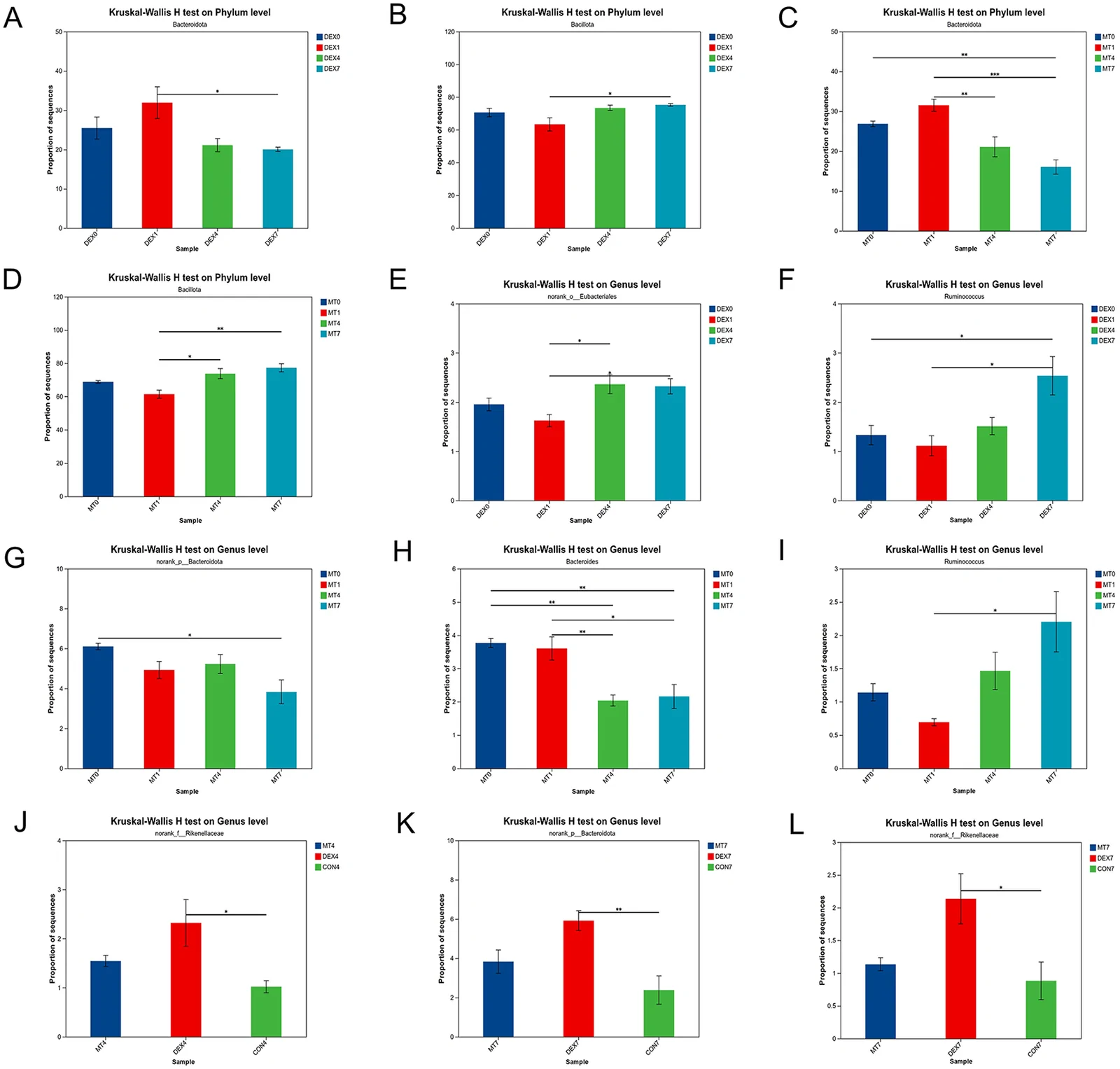
Effects of transport on the gut microbiota of cattle at the phylum and genus levels. **(A, B)** *Bacteroidota* and *Bacillota* in the DEX group. **(C, D)** *Bacillota* and *Bacteroidota* in the MT group. **(E, F)** *norank_o__Eubacteriales, Ruminococcus* in the DEX group. **(G–I)** *norank_p__Bacteroidota, Bacteroides, Ruminococcus* in the MT group. **(J)** *norank_f__Rikenellaceae* on Day 4 post-transport across the groups. **(K, L)** *norank_p_Bacteroidota, norank_f__Rikenellaceae* on Day 7 post-transport across the groups. CON0 indicates the CON group before transport; CON1, CON4, and CON7 indicate day 1, day 4, and day 7 after arrival, respectively. The same labeling applies to the MT and DEX groups. *P < 0.05, **P < 0.01, ***P < 0.001.

At the genus level, in the DEX group, *Ruminococcus* at D7 was significantly higher than at D0 and D1 (*P* < 0.05), and *norank_o__Eubacteriales* at D4 was significantly higher than at D1 (*P* < 0.05) (Figures 7E, F). In the MT group, *norank_p__Bacteroidota* at D7 was significantly lower than at D0 (*P* < 0.05), *Bacteroides* at both D4 and D7 was significantly lower than at D0 (*P* < 0.01); moreover, compared with D1, *Bacteroides* decreased significantly at D4 (*P* < 0.01) and remained significantly lower at D7 (*P* < 0.05), and *Ruminococcus* at D7 was significantly higher than at D1 (*P* < 0.05) (Figures 7G–I). At D4, the relative abundance of *norank_f__Rikenellaceae* in the DEX4 group was significantly higher than that in CON4 (*P* < 0.05) (Figure 7J). At D7, the relative abundance of *norank_p__Bacteroidota* in DEX7 was significantly higher than that in CON7 (*P* < 0.01), and *norank_f__Rikenellaceae* in DEX7 was also significantly higher than in CON7 (*P* < 0.05) (Figures 7K–L).

#### 3.4.5 Functional prediction

The COG functional classification analysis showed that, before transport, the predicted functions of the fecal microbiota were mainly enriched in fundamental categories, including amino acid transport and metabolism (E), translation and ribosomal structure (J), transcription (K), cell wall/membrane/envelope biogenesis (M), and energy production and conversion (C). In contrast, functions related to the cytoskeleton, chromatin structure and dynamics, RNA processing and modification, and extracellular structures (Z, B, A, and W) were present at relatively low abundances (Figure 8A). At D1, D4, and D7, the overall ranking and distribution patterns of COG functional categories were largely consistent with those at D0, showing only minor fluctuations without marked increases or decreases in any functional block. This suggests that, under the conditions of the present study, the overall functional potential of the gut microbiota remained relatively stable at the COG level. Only a few categories (e.g., energy production and conversion) showed statistically significant differences between specific time points and treatment groups; however, the absolute magnitude of these changes was small, indicating that transport and the interventions may have only a modest influence on energy metabolism–related functions (Figures 8B–D).

**Figure 8:**
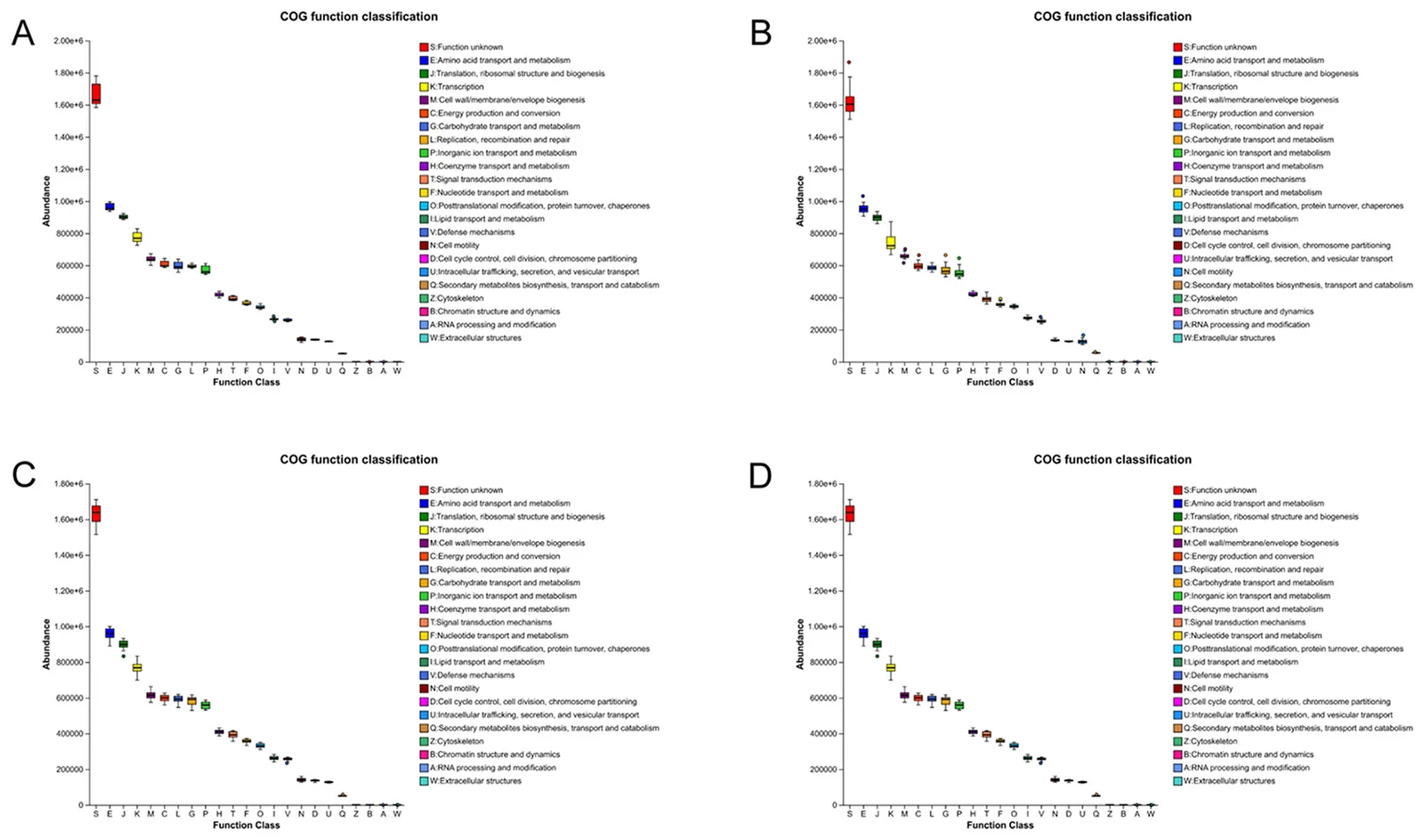
PICRUSt2-based prediction of COG functional categories of the fecal microbiota. **(A)** Before transport (D0); **(B)** day 1 after transport (D1); **(C)** day 4 after transport (D4); **(D)** day 7 after transport (D7). The X-axis represents different COG functional categories, with each letter corresponding to a specific functional class. The Y-axis represents the gene abundance associated with each functional category. Different colored boxplots indicate the distribution of gene abundance for each functional class across samples. The box represents the interquartile range, the line inside the box indicates the median, and the whiskers represent the dispersion of the data.

## 4 Discussion

This study aimed to investigate the effects of melatonin and dexmedetomidine administration on the physiological status and gut microbiota of Simmental cattle after transport stress. Transport stress induced alterations in hematological parameters and gut microbiota (38), while melatonin and dexmedetomidine alleviated these stress responses to a certain extent. This study has several limitations, including the relatively small sample size and the evaluation of the effects of melatonin and dexmedetomidine only under a 6 h transportation stress condition. Future studies should include larger sample sizes and investigate the effects of these interventions under different transportation durations to improve the reliability and robustness of the findings.

Previous studies have demonstrated that transportation stress typically peaks within 24 h post-arrival (39), characterized by heightened immune responses, and other physiological disruptions (40). Although these responses tend to subside over the subsequent days, recovery trajectories vary depending on species, transport conditions, and individual resilience (41). In healthy animals, stress-induced alterations in metabolic and immune function generally resolve within one week (42). However, excessive stress intensity or prolonged exposure may result in chronic stress or immunosuppression, with potentially long-lasting adverse effects on animal health (43).

### 4.1 Effects of transportation stress on cattle blood parameters

The hematological findings of the present study indicate that several parameters were significantly altered following transport-induced stress. Specifically, RBC and HGB were significantly increased in the MT group at D7, suggesting that melatonin may facilitate a more rapid recovery of erythropoiesis after transport. This observation is consistent with previous studies reporting that melatonin can significantly increase erythrocyte count and hemoglobin levels (44, 45). Although no significant treatment × time interaction effects were detected for WBC, NEU, and MON, significant time effects were observed. Notably, the changes in these parameters were more moderate in the MT and DEX groups, with levels at D7 remaining lower than those in the CON group. Previous studies have demonstrated that melatonin can attenuate stress-induced alterations in immune cells and suppress inflammatory responses (46), whereas dexmedetomidine reduces stress levels by inhibiting excessive sympathetic nervous system activity, thereby limiting abnormal immune cell fluctuations (47). These mechanisms may account for the relatively attenuated changes in inflammation-related cells observed in the MT and DEX groups. Lymphocytes and eosinophils showed no significant changes under the present experimental conditions, suggesting that these cell populations may exhibit relative stability in response to transport stress of this intensity.

In terms of plasma biochemical parameters, Glu-G levels in the CON group were higher than those in the MT and DEX groups. Transport-induced activation of the neuroendocrine system is known to trigger stress-related hyperglycemia. The regulatory effect of melatonin on excessive activation of the HPA axis (9), together with the stress-attenuating effect of dexmedetomidine mediated through central sedation (27), may account for the lower glucose levels observed in these two groups. These findings also suggest a potential role of both agents in alleviating disturbances in glucose metabolism. Previous studies have shown that AST and ALT levels increase rapidly in response to muscle or hepatic injury (48). A similar pattern was observed in the present study, with AST and ALT levels peaking at D1 following transport and subsequently declining over time. No significant changes were observed in TP or ALB, suggesting that, under the present experimental conditions, transport did not markedly disrupt the homeostasis of these parameters in Simmental cattle.

Transport stress can also induce oxidative stress, which is characterized by increased free radical production and altered antioxidant enzyme activity. Superoxide dismutase and catalase play critical roles in scavenging free radicals and reactive oxygen species (ROS), thereby mitigating oxidative damage (49). Previous studies have reported significant alterations in SOD and CAT activities following transport, such as increased SOD activity accompanied by decreased CAT activity (6, 50), which may contribute to maintaining cellular defense capacity and antioxidant protection (51). In the present study, a significant time × treatment interaction was observed for SOD activity. On D7, SOD activity was significantly higher in both the MT and DEX groups than in the CON group, whereas the CON group exhibited significantly lower SOD activity than on D1 and D4, suggesting that MT and DEX may contribute to maintaining antioxidant capacity during the post-transport recovery period. In contrast, no significant time × treatment interaction was observed for CAT activity, although significant effects of time and treatment were detected. CAT activity increased on D4 compared with D1 but decreased on D7 relative to both D1 and D4, indicating that CAT activity underwent dynamic changes during post-transport recovery. Overall, under the conditions of this study, MT and DEX treatments did not result in abnormal suppression of antioxidant enzyme activity. The relatively higher SOD levels observed at D7 further indicate that these two interventions may exert beneficial effects on recovery from transport-induced oxidative stress.

Transportation stress can induce alterations in endocrine responses, among which ACTH and COR are commonly used indicators of the stress response (52). However, because hormone secretion is regulated by multiple factors, including feedback mechanisms, circadian rhythms, and tissue sensitivity, ACTH and COR may exhibit distinct patterns during the post-transport recovery period (53). In the present study, a significant time × treatment interaction was observed for ACTH. ACTH concentrations in the MT group were lower on D4 than on D1, whereas ACTH concentrations in the DEX group were increased on D7. As ACTH secretion is regulated by multiple physiological factors, these changes may reflect dynamic adjustments in pituitary regulatory activity during the recovery process rather than serve as an independent indicator of changes in stress status (54). In contrast to ACTH, no significant time × treatment interaction was observed for COR. Instead, a significant effect of time was detected, with COR concentrations on D7 being lower than those on D1 and D4. These findings suggest that changes in COR were primarily associated with the temporal progression of post-transport recovery, whereas MT and DEX did not markedly alter its recovery pattern. Previous studies have shown that transportation stress can increase cortisol concentrations in beef cattle, whereas physiological indicators gradually recover following the cessation of transportation stress and progressive adaptation of the animals (55). Therefore, the decline in COR over time observed in the present study may reflect the transition from an acute stress response to a recovery state following transportation.

### 4.2 Effects of transportation stress on the gut microbiota of cattle

The gut microbiota plays a vital role in maintaining host health, with growing evidence supporting its involvement in immune regulation, metabolic homeostasis, neural function, and disease prevention (56). In animals, the diversity of the gastrointestinal microbiota is closely associated with metabolic efficiency, nutrient absorption, and immune competence (57). Microbial abundance is essential for sustaining the dynamic equilibrium of the intestinal ecosystem, thereby exerting a direct influence on overall health and growth performance (58). Conversely, reductions in microbial diversity and richness may disrupt this balance, potentially leading to adverse health outcomes (59).

The results of the present study indicate that transportation stress altered the gut microbiota structure of Simmental cattle. In the CON group, the Shannon index was significantly reduced on D7 after transportation, whereas it remained relatively stable in both the MT and DEX groups. Moreover, β-diversity analysis showed a clear separation in gut microbial composition among the three groups, indicating that both interventions were associated with changes in gut microbiota composition following transportation. These effects are consistent with previous studies reporting that melatonin promotes remodeling of the intestinal microbial community, suppresses oxidative stress, reduces autophagy and inflammatory responses, and increases the abundance of beneficial bacteria and the production of short-chain fatty acids, thereby improving gut health (60). In addition, dexmedetomidine intervention has been shown to alter gut microbiota composition in mice exposed to acute stress and to help maintain intestinal microbial stability (61).

In the present study, *Bacillota* and *Bacteroidota* were identified as the dominant phyla in all groups, which is consistent with previous reports on the bovine gut microbiota (38). Following transportation, the relative abundance of *Bacillota* increased significantly, whereas that of *Bacteroidota* decreased significantly in the DEX group. A similar trend was also observed in the MT group, suggesting that both treatments were associated with alterations in gut microbiota composition following transportation. As core members of the gastrointestinal microbiota in ruminants, *Bacillota* and *Bacteroidota* play vital roles in digestion, nutrient absorption, energy metabolism, and in protecting the host against enteric pathogens (62). The ratio of *Bacillota* to *Bacteroidota* is generally considered to reflect the metabolic potential of the gut microbiota and can modulate host metabolism, physiology, and health. *Bacillota* promote the degradation of dietary fibers, converting cellulose into volatile fatty acids, thereby facilitating food digestion and growth, whereas *Bacteroidota* primarily enhance the digestion and absorption of dietary proteins and carbohydrates, contributing to the development of the gastrointestinal immune system (63). *Bacteroidota* are capable of metabolizing starch, non-cellulosic polysaccharides, and monosaccharides, with succinate being their primary fermentation product. In the rumen, succinate is decarboxylated to propionate, which is essential for maintaining glucose homeostasis in the host animal (64).

At the genus level, the relative abundance of *Ruminococcus* was significantly increased during the late recovery stage (D7) in both the DEX and MT groups. Previous studies have shown that certain members of the genus *Ruminococcus* are involved in the degradation of complex carbohydrates and plant-derived polysaccharides and are commonly recognized as fiber degrading bacteria in the gastrointestinal tract of ruminants (65). Therefore, the increased abundance of *Ruminococcus* observed in the present study suggests that this genus underwent dynamic changes during post-transport recovery, although whether these changes were accompanied by alterations in metabolic function requires further investigation. Meanwhile, the relative abundance of *Bacteroides* in the MT group continuously decreased throughout the recovery period. In addition, the relative abundance of *norank_p__Bacteroidota* on D7 was lower than that on D0. As a major member of the phylum *Bacteroidota, Bacteroides* is widely involved in the utilization of complex polysaccharides (66). Its altered abundance further indicates that MT treatment was associated with changes in gut microbiota composition. Furthermore, in the between-group comparison, the relative abundance of *norank_f__Rikenellaceae* was higher in the DEX group than in the CON group on D4, whereas the relative abundances of both *norank_p__Bacteroidota* and *norank_f__Rikenellaceae* were higher in the DEX group than in the CON group on D7, suggesting that dexmedetomidine treatment was associated with changes in the composition of specific *Bacteroidota*-related taxa. In addition, the relative abundance of *norank_o__Eubacteriales* was higher in the DEX group on D4 and D7 than on D1, further indicating that the gut microbiota composition underwent continuous dynamic changes during post-transport recovery.

The results of the present study indicate that the gut microbiota composition of cattle underwent dynamic changes following transportation, and that both MT and DEX influenced changes in specific dominant taxa. However, gut microbiota composition was analyzed using 16S rRNA gene sequencing, and the functional predictions generated by PICRUSt2 were inferred solely from reference genome information rather than direct measurements of microbial metabolic activity. Therefore, the discussion of gut microbiota alterations in the present study was primarily based on the known characteristics of the relevant taxa reported in previous studies, and their biological significance requires further validation through metagenomic and metabolomic analyses, together with host-related functional indicators.

## 5 Conclusions

The present study demonstrated that transportation significantly affected physiological parameters and gut microbiota composition in beef cattle. Notably, SOD activity was significantly higher in both the MT and DEX groups than in the CON group on D7. In addition, α-diversity of the gut microbiota was significantly reduced in the CON group following transportation, whereas it remained relatively stable in the MT and DEX groups. Both interventions were associated with changes in gut microbiota structure and the relative abundance of specific bacterial taxa, suggesting that MT and DEX may influence gut microbiota composition and its recovery following transportation. However, microbial metabolites and gut function-related indicators were not directly assessed in the present study. Therefore, the biological significance of these microbial alterations requires further investigation.

## Data Availability

The original contributions presented in the study are publicly available. This data can be found in the NCBI repository: https://www.ncbi.nlm.nih.gov/sra/?term=PRJNA1526045.
