# Supplementary material for: Physiological responses and gut microbiota dynamics in beef cattle following transport stress syndrome with melatonin or dexmedetomidine administration
Source: Front Vet Sci. 2026 Sep 21;13:1861919. doi: 10.3389/fvets.2026.1861919 (PMC13637517; doi:10.3389/fvets.2026.1861919)
Supplement: Supplementary file 1 [file Table_1.docx]

**Supplementary Table 1.** Coefficients of variation for hematological parameters in transported Simmental cattle across different treatment groups

| Indicators | Time Point | Group | | |
| --- | --- | --- | --- | --- |
|  |  | CON | MT | DEX |
| WBC (%) | D0 | 21.43 | 25.86 | 11.20 |
|  | D1 | 19.24 | 42.92 | 25.02 |
|  | D4 | 22.18 | 28.95 | 35.36 |
|  | D7 | 48.51 | 27.18 | 18.96 |
| NEU (%) | D0 | 23.91 | 38.71 | 27.80 |
|  | D1 | 30.86 | 44.97 | 27.84 |
|  | D4 | 31.06 | 48.88 | 72.77 |
|  | D7 | 81.19 | 57.03 | 42.41 |
| LYM (%) | D0 | 27.96 | 25.59 | 5.31 |
|  | D1 | 15.09 | 49.76 | 28.90 |
|  | D4 | 20.34 | 19.59 | 16.80 |
|  | D7 | 26.21 | 13.20 | 21.54 |
| MON (%) | D0 | 55.33 | 26.90 | 53.24 |
|  | D1 | 38.05 | 64.08 | 44.41 |
|  | D4 | 52.51 | 24.97 | 61.94 |
|  | D7 | 40.53 | 17.85 | 20.56 |
| EOS (%) | D0 | 78.45 | 42.41 | 54.01 |
|  | D1 | 55.81 | 44.97 | 54.15 |
|  | D4 | 53.41 | 64.97 | 55.68 |
|  | D7 | 68.97 | 57.19 | 50.31 |
| RBC (%) | D0 | 5.72 | 5.48 | 12.16 |
|  | D1 | 9.56 | 17.60 | 10.41 |
|  | D4 | 5.83 | 11.51 | 12.30 |
|  | D7 | 14.98 | 10.87 | 5.84 |
| HGB (%) | D0 | 7.45 | 6.38 | 11.00 |
|  | D1 | 3.78 | 16.17 | 6.03 |
|  | D4 | 5.42 | 4.46 | 8.11 |
|  | D7 | 10.54 | 7.37 | 2.55 |
| MCH (%) | D0 | 3.27 | 7.35 | 3.18 |
|  | D1 | 5.81 | 8.25 | 5.79 |
|  | D4 | 5.36 | 8.38 | 5.50 |
|  | D7 | 6.21 | 9.23 | 6.32 |
| PLT (%) | D0 | 88.21 | 22.86 | 65.43 |
|  | D1 | 26.39 | 62.94 | 77.68 |
|  | D4 | 36.07 | 28.22 | 61.65 |
|  | D7 | 75.01 | 68.94 | 87.35 |

Note: the treatment groups were CON, MT, and DEX; accordingly, CON, MT, and DEX denote the control group, melatonin-treated group, and dexmedetomidine-treated group (n = 5), respectively. D0 indicates pre-transport, whereas D1, D4, and D7 indicate days 1, 4, and 7 after transport, respectively. WBC, white blood cells; NEU, neutrophils; LYM, lymphocytes; MON, monocytes; EOS, eosinophils; RBC, red blood cells; HGB, hemoglobin; MCH, mean corpuscular hemoglobin; PLT, platelets.
