# Supplementary material for: Physiological responses and gut microbiota dynamics in beef cattle following transport stress syndrome with melatonin or dexmedetomidine administration
Source: Front Vet Sci. 2026 Sep 21;13:1861919. doi: 10.3389/fvets.2026.1861919 (PMC13637517; doi:10.3389/fvets.2026.1861919)
Supplement: Supplementary file 2 [file Table_2.docx]

**Supplementary Table 2.** Coefficients of variation for plasma biochemical parameters in transported Simmental cattle across different treatment groups

| Indicators | Time Point | Group | | |
| --- | --- | --- | --- | --- |
|  |  | CON | MT | DEX |
| Glu-G (%) | D0 | 4.03 | 3.79 | 4.01 |
|  | D1 | 12.83 | 16.23 | 8.36 |
|  | D4 | 15.10 | 10.11 | 9.62 |
|  | D7 | 16.84 | 8.43 | 2.78 |
| AST (%) | D0 | 20.99 | 16.93 | 12.96 |
|  | D1 | 24.82 | 29.60 | 25.49 |
|  | D4 | 12.67 | 32.65 | 9.46 |
|  | D7 | 17.70 | 17.85 | 20.16 |
| ALT (%) | D0 | 9.67 | 15.44 | 21.80 |
|  | D1 | 10.92 | 15.14 | 23.32 |
|  | D4 | 15.31 | 20.83 | 14.28 |
|  | D7 | 11.18 | 16.58 | 16.08 |
| TP (%) | D0 | 4.25 | 3.23 | 3.41 |
|  | D1 | 8.80 | 6.30 | 5.20 |
|  | D4 | 8.43 | 7.44 | 7.64 |
|  | D7 | 12.90 | 5.73 | 3.25 |
| ALB (%) | D0 | 6.01 | 8.01 | 8.13 |
|  | D1 | 6.05 | 4.03 | 4.40 |
|  | D4 | 10.53 | 9.95 | 6.54 |
|  | D7 | 8.28 | 5.91 | 4.18 |

Note: the treatment groups were CON, MT, and DEX; accordingly, CON, MT, and DEX denote the control group, melatonin-treated group, and dexmedetomidine-treated group (n = 5), respectively. D0 indicates pre-transport, whereas D1, D4, and D7 indicate days 1, 4, and 7 after transport, respectively. Glu-G, glucose; ALT, alanine aminotransferase; AST, aspartate aminotransferase; TP, total protein; ALB, albumin.
