# Supplementary material for: Physiological responses and gut microbiota dynamics in beef cattle following transport stress syndrome with melatonin or dexmedetomidine administration
Source: Front Vet Sci. 2026 Sep 21;13:1861919. doi: 10.3389/fvets.2026.1861919 (PMC13637517; doi:10.3389/fvets.2026.1861919)
Supplement: Supplementary file 3 [file Table_3.docx]

**Supplementary Table 3.** Coefficients of variation for plasma hormone and oxidative stress-related parameters in transported Simmental cattle across different treatment groups

| Indicators | Time Point | Group | | |
| --- | --- | --- | --- | --- |
|  |  | CON | MT | DEX |
| COR (%) | D0 | 30.08 | 8.17 | 9.61 |
|  | D1 | 22.92 | 14.06 | 19.66 |
|  | D4 | 21.17 | 11.34 | 20.93 |
|  | D7 | 41.57 | 12.64 | 25.68 |
| ACTH (%) | D0 | 12.42 | 15.62 | 11.32 |
|  | D1 | 14.60 | 13.63 | 11.13 |
|  | D4 | 18.81 | 14.90 | 10.58 |
|  | D7 | 14.25 | 22.24 | 6.81 |
| SOD (%) | D0 | 11.39 | 15.85 | 26.76 |
|  | D1 | 11.15 | 15.92 | 15.54 |
|  | D4 | 40.49 | 30.83 | 43.16 |
|  | D7 | 46.29 | 13.30 | 20.64 |
| CAT (%) | D0 | 10.37 | 16.06 | 10.89 |
|  | D1 | 15.10 | 9.72 | 15.04 |
|  | D4 | 12.51 | 10.75 | 14.49 |
|  | D7 | 23.20 | 14.88 | 15.33 |

Note: the treatment groups were CON, MT, and DEX; accordingly, CON, MT, and DEX denote the control group, melatonin-treated group, and dexmedetomidine-treated group (n = 5), respectively. D0 indicates pre-transport, whereas D1, D4, and D7 indicate days 1, 4, and 7 after transport, respectively. COR, cortisol; ACTH, adrenocorticotropic hormone; SOD, superoxide dismutase; CAT, catalase.
