# Supplementary material for: Physiological responses and gut microbiota dynamics in beef cattle following transport stress syndrome with melatonin or dexmedetomidine administration
Source: Front Vet Sci. 2026 Sep 21;13:1861919. doi: 10.3389/fvets.2026.1861919 (PMC13637517; doi:10.3389/fvets.2026.1861919)
Supplement: Supplementary file 4 [file Table_4.docx]

**Supplementary Table 4.** Baseline comparison of hematological parameters in transported Simmental cattle before transport (D0) among different treatment groups

| Indicators | Group | | | *P* value |
| --- | --- | --- | --- | --- |
|  | CON | MT | DEX |  |
| WBC (10^9/L) | 7.18±1.54 | 9.87±2.55 | 7.38±0.83 | 0.06 |
| NEU (10^9/L) | 2.16±0.52 | 3.32±1.29 | 2.32±0.64 | 0.12 |
| LYM (10^9/L) | 4.59±1.28 | 6.18±1.58 | 4.53±0.24 | 0.08 |
| MON (10^9/L) | 0.30±0.17 | 0.22±0.06 | 0.27±0.15 | 0.65 |
| EOS (10^9/L） | 0.13±0.10 | 0.14±0.06 | 0.26±0.14 | 0.15 |
| RBC (10^12/L) | 7.03±0.40 | 7.44±0.41 | 7.25±0.88 | 0.57 |
| HGB (g/L) | 107.00±7.97 | 108.00±6.89 | 102.60±11.28 | 0.61 |
| MCH (pg) | 15.22±0.50 | 14.58±1.07 | 14.16±0.45 | 0.11 |
| PLT (10^9/L) | 179.00±157.90 | 304.20±69.54 | 175.20±114.64 | 0.19 |

Note: data are presented as mean ± standard deviation (SD). All parameters were measured before transport (D0) and were used to compare baseline differences among treatment groups. The treatment groups were CON, MT, and DEX; accordingly, CON, MT, and DEX denote the control group, melatonin-treated group, and dexmedetomidine-treated group (n = 5), respectively. *P* values represent the overall between-group comparisons for each parameter among the CON, MT, and DEX groups at D0. A value of *P* < 0.05 was considered statistically significant. WBC, white blood cells; NEU, neutrophils; LYM, lymphocytes; MON, monocytes; EOS, eosinophils; RBC, red blood cells; HGB, hemoglobin; MCH, mean corpuscular hemoglobin; PLT, platelets.
