# Supplementary material for: Physiological responses and gut microbiota dynamics in beef cattle following transport stress syndrome with melatonin or dexmedetomidine administration
Source: Front Vet Sci. 2026 Sep 21;13:1861919. doi: 10.3389/fvets.2026.1861919 (PMC13637517; doi:10.3389/fvets.2026.1861919)
Supplement: Supplementary file 5 [file Table_5.docx]

**Supplementary Table 5.** Baseline comparison of plasma biochemical parameters in transported Simmental cattle before transport (D0) among different treatment groups

| Indicators | Group | | | *P* value |
| --- | --- | --- | --- | --- |
|  | CON | MT | DEX |  |
| Glu-G (mmol/L) | 4.33±0.17 | 4.32±0.16 | 4.27±0.17 | 0.80 |
| ALT (U/L) | 26.58±2.57 | 24.54±3.79 | 25.84±5.63 | 0.74 |
| AST (U/L) | 83.84±17.60 | 82.84±14.03 | 88.82±11.51 | 0.79 |
| TP (g/L) | 67.32±2.86 | 67.22±2.17 | 64.00±2.19 | 0.09 |
| ALB (g/L) | 28.78±1.73 | 29.80±2.39 | 28.86±2.35 | 0.72 |

Note: data are presented as mean ± SD. All parameters were measured before transport (D0) and were used to compare baseline differences among treatment groups. The treatment groups were CON, MT, and DEX; accordingly, CON, MT, and DEX denote the control group, melatonin-treated group, and dexmedetomidine-treated group (n = 5), respectively. *P* values represent the overall between-group comparisons for each parameter among the CON, MT, and DEX groups at D0. A value of *P* < 0.05 was considered statistically significant. Glu-G, glucose; ALT, alanine aminotransferase; AST, aspartate aminotransferase; TP, total protein; ALB, albumin.
