# Supplementary material for: Physiological responses and gut microbiota dynamics in beef cattle following transport stress syndrome with melatonin or dexmedetomidine administration
Source: Front Vet Sci. 2026 Sep 21;13:1861919. doi: 10.3389/fvets.2026.1861919 (PMC13637517; doi:10.3389/fvets.2026.1861919)
Supplement: Supplementary file 6 [file Table_6.docx]

**Supplementary Table 6.** Baseline comparison of plasma hormone and oxidative stress-related parameters in transported Simmental cattle before transport (D0) among different treatment groups

| Indicators | Group | | | *P* value |
| --- | --- | --- | --- | --- |
|  | CON | MT | DEX |  |
| COR (pg/mL) | 198.98±59.85 | 252.69±20.65 | 260.80±25.05 | 0.06 |
| ACTH (pg/mL) | 285.76±35.49 | 253.21±39.56 | 281.60±31.88 | 0.33 |
| SOD (pg/mL) | 360.74±41.10 | 228.23±36.19 | 330.53±88.47 | 0.01 |
| CAT (pg/mL) | 367.19±38.09 | 379.97±61.02 | 390.94±42.57 | 0.74 |

Note: data are presented as mean ± SD. All parameters were measured before transport (D0) and were used to compare baseline differences among treatment groups. The treatment groups were CON, MT, and DEX; accordingly, CON, MT, and DEX denote the control group, melatonin-treated group, and dexmedetomidine-treated group (n = 5), respectively. *P* values represent the overall between-group comparisons for each parameter among the CON, MT, and DEX groups at D0. A value of *P* < 0.05 was considered statistically significant. COR, cortisol; ACTH, adrenocorticotropic hormone; SOD, superoxide dismutase; CAT, catalase.
